# Comprehensive Analysis of Nivolumab, A Therapeutic Anti-Pd-1 Monoclonal Antibody: Impact of Handling and Stress

**DOI:** 10.3390/pharmaceutics14040692

**Published:** 2022-03-23

**Authors:** Anabel Torrente-López, Jesús Hermosilla, Antonio Salmerón-García, José Cabeza, Natalia Navas

**Affiliations:** 1 Department of Analytical Chemistry, Science Faculty, Biohealth Research Institute (ibs.GRANADA), University of Granada, E-18071 Granada, Spain; anabeltl@ugr.es (A.T.-L.); jesushf@ugr.es (J.H.); 2 Department of Clinical Pharmacy, Biohealth Research Institute (ibs.GRANADA), San Cecilio University Hospital, E-18012 Granada, Spain; asalgar6@gmail.com (A.S.-G.); jose.cabeza.sspa@juntadeandalucia.es (J.C.)

**Keywords:** nivolumab, comprehensive analytical characterisation, forced degradation, UHPLC-MS, isoform profile

## Abstract

Nivolumab, formulated in the medicine Opdivo^®^ (10 mg/mL), is a therapeutic monoclonal antibody (mAb) used in the treatment of different types of cancer. Currently, there is insufficient knowledge about the behaviour of this protein with regards to the risk associated with its routine handling or unintentional mishandling, or when subjected to stress conditions in hospitals. These conditions can be simulated in forced degradation studies, which provide an in-depth understanding of the biophysical and biochemical properties of mAbs. In this study, we carried out a physicochemical and functional characterisation of nivolumab, which was subjected to various stress conditions: heat, freeze/thaw cycles, agitation, light exposure and high hypertonic solution. We used a wide range of analytical techniques: Far-UV CD, IT-FS, DLS, SE/UHPLC(UV)-[Native]MS, and ELISA. The results show that exposure to light was the stress test with the greatest impact on the samples, revelling the formation of non-natural dimers and a different isoform profile. In addition, nivolumab (Opdivo^®^) demonstrated stability up to 60 °C (1 h). As regards functionality all the nivolumab (Opdivo^®^) stressed samples were found to be stable except for those subjected to light and agitation, and to a lesser extent, those subjected to FTC 5 and NaCl stresses.

## 1. Introduction

Therapeutic monoclonal antibodies (mAbs) are a major class of biopharmaceuticals which have seen rapid growth in recent decades. They have an immunoglobulin (Ig) structure that is capable of specific binding to unique epitopes present in their target. As a consequence, mAbs have notably improved the treatment results for a wide variety of human-specific diseases such as cancer, autoimmune diseases, cardiovascular disorders, ophthalmic diseases, or asthma [1]. Most approved mAbs are selected from three human IgG isotypes (IgG1, IgG2, or IgG4), which are defined by the total number of disulphide bridges (16 for IgG1 and IgG4, and 18 for IgG2), disulphide bonds in the flexible hinge region (two for IgG1 and IgG4, and four for IgG2) and the different heavy-chain amino acid sequences. At present, the majority of mAbs that have been approved by the FDA and EMA are based on the IgG1 isotype, while IgG3-based therapeutics have received little attention for therapy development owing to their short half-life in the body, given that the clearance rate of IgG3 is significantly faster than that of other isotypes [2,3]. As for the IgG4 isotype, there has been a progressive increase in the number of IgG4-based therapeutics on the market and in clinical trials. This is thanks to the remarkable progress that biotechnological and pharmaceutical companies have made in developing more stabilised IgG4 formats, such as by introducing S228P point mutation in the core-hinge sequence of the mAb [2]. One of the therapeutic mAbs that is manufactured using hinge-stabilised engineering is nivolumab (Opdivo^®^ from Bristol-Myers Squibb, Dublin, Ireland), a human IgG4 mAb, which binds to the programmed death-1 (PD-1) receptor and blocks its interaction with the ligands PD-L1 and PD-L2, according to the Summary of Product Characteristics for Opdivo^®^ issued by the European Medicine Agency [4]. By blocking this pathway, nivolumab potentiates T-cell responses, including anti-tumour responses [5]. This is why this mAb is used, either by itself or in combination with another mAb, i.e., ipilimumab or cabozantinib, in cancer therapy. Opdivo^®^ is indicated for the treatment of melanoma; non-small cell lung cancer (NSCLC); malignant pleural mesothelioma (MPM); renal cell carcinoma (RCC); and classical Hodgkin lymphoma (cHL), among other types of cancer. Nivolumab is produced in Chinese Hamster Ovary (CHO) cells using recombinant DNA technology and is presented as a concentrate for solution for infusion (sterile concentrate) at 10 mg/mL [4].

Like all mAbs, nivolumab consists of a large, highly complex molecule and is extremely sensitive to its environment [6]. Before administration to patients, mAbs may be subject to environmental stress conditions such as exposure to light, and mechanical or thermal stresses, among others [7,8]. These stresses can have a negative impact on the structure of the protein, leading to its chemical or physical degradation [9,10,11,12]. Routine handling prior to administration—e.g., for sample preparation—or inadvertent mishandling—e.g., incorrect storage—can expose mAbs to these environmental stress conditions, so giving rise to the aforementioned forms of degradation [8]. These forms of degradation alter the quality of mAb-based products and can have a significant effect on their therapeutic efficacy, by limiting their bioactivity or increasing their immunogenicity [6,7]. One of the main concerns with therapeutic proteins is the increase of aggregation in clinical samples, a form of degradation that could modify the ability of the mAb to correctly interact with its specific target, and could lead to immunogenicity. Detecting degraded mAbs before administration to patients is therefore crucial for ensuring high quality, efficacy, and safety [7].

Forced degradation studies offer an opportunity to gain an in-depth understanding of the biophysical and biochemical properties of mAbs [13]. They can provide valuable information on the degradation pathways to which mAbs are exposed during handling in hospitals prior to administration. Usually, these studies involve subjecting a particular biopharmaceutical product to a range of relatively harsh experimental stress conditions for a short period of time. It is important to avoid applying both too much or too little stress. The information that forced degradation studies provide is very useful in support of real-time environmental conditions and when dealing with practical problems, such as when there is a break in the cold chain; when making pharmaceutical preparations prior to administration; or when deciding whether to use the surplus product from one treatment on another patient [13,14,15,16]. The forced degradation conditions employed in such studies typically include high temperature; freeze-thaw cycles; agitation; light; high ionic strength exposure, among others. These conditions are selected according to the probability that the medicine may be exposed to them during the different stages of handling these products in hospital. The most common degradation pathways are aggregation, fragmentation, deamidation and oxidation [13].

Forced degradation of nivolumab (Opdivo^®^)—as a representative example of an IgG4—[7] was conducted to evaluate the influence of degraded mAbs in a previously reported flow injection analysis (FIA) combined with UV spectroscopy and a statistical matching method [17] for the quality control of compounded mAbs in hospital. Surprisingly, when this FIA strategy was initially proposed, it was not validated by checking the specificity, although the method was proposed for mAb quantification. As a result, the stress study conducted later on nivolumab focused more on validating the FIA methodology applied in the hospital quality control process than on presenting a detailed study of the degradation of nivolumab. It did not assess, for example, the functionality of degraded nivolumab. Within the context of a long-term stability study, nivolumab was studied in its original vials after opening and handling in a normal saline bag for intravenous infusion [14]. Several physicochemical and functional properties of nivolumab were analysed. Although this research presented very valuable results, it did not focus on degradation studies, as its main objective was to study the stability of nivolumab over a period of more than 24 h, at different temperatures and after freeze/thaw cycles.

The aim of this research is therefore to carry out a comprehensive analytical characterisation and a forced degradation study of nivolumab (Opdivo^®^ 10 mg/mL). For a proper characterisation of mAbs, complementary techniques must be used, including an appropriate set of physicochemical and functional tests. For this reason, we used a wide range of techniques to carry out this study, such as circular dichroism (CD) for the analysis of the secondary structure; fluorescence for the assessment of the tertiary structure; dynamic light scattering (DLS) to track particulate in the solutions; size-exclusion chromatography with UV detection (SE/UHPLC(UV)) to analyse oligomers; size-exclusion chromatography coupled to mass spectrometry (SE/UHPLC(UV)-MS(Orbitrap)) to determine the isoform profile including glycans; and an Enzyme-Linked Immunosorbent Assay (ELISA) to evaluate functionality by means of the specific bind nivolumab-PD1. To this end, forced degradation tests such as heating, freeze/thaw, agitation, light exposure and high ionic strength stresses were performed on samples of the medicine to evaluate degradation. These tests produced very interesting results that provide valuable new information about this biopharmaceutical, which could be useful for evaluating the potential consequences associated with its routine handling or unintentional mishandling in hospital.

## 2. Materials and Methods

### 2.1. Nivolumab Samples

Opdivo^®^ vials (nivolumab 10 mg/mL, Bristol-Myers Squibb Pharma EEIG, Dublin, Ireland) were kindly supplied for this study by the Pharmacy Unit of the University Hospital “San Cecilio” (Granada, Spain). Hospital leftovers were used throughout the study and the analyses were performed immediately after opening the vial to ensure the stability indicated by the manufacturer [4]. The following batches were used during this study: ABJ9188; ABK3907; ABR9823; ABS8408; ABV0452; ABV2650; and ABW3154.

### 2.2. Forced Degradation (Stresses)

Forced degradation studies were carried out on the medicine Opdivo^®^ by exposing the samples to each particular stress condition, always within the expiry date of the medicine once opened in order to avoid any degradation in nivolumab caused by the passage of time. Seven forced degradation conditions were tested: (i) exposure to a temperature of 40 °C for 1 h in a ThermoMixer^®^ C thermoblock 1.5 mL (Eppendorf, Hamburg, Germany); (ii) exposure to a temperature of 60 °C for 1 h in a ThermoMixer^®^ C thermoblock 1.5 mL (Eppendorf, Hamburg, Germany); (iii) one freeze–thaw cycle (room temperature of around 21/25 °C to −20 °C) in a vertical Bosch freezer (GSE32420, Gerlingen, Germany); (iv) five freeze–thaw cycles (room temperature of around 21/25 °C to −20 °C in a vertical Bosch freezer (GSE32420, Gerlingen, Germany); (v) exposure to light irradiation (250 W/m^2^) for 24 h in an aging chamber (Solarbox 3000e RH, Cofomegra, Milan, Italy); (vi) agitation (300 rounds/min) for 24 h in a mechanical laboratory shaker (type 3006, Gesellschaft für Labortechnik, Burgwedel, Germany); and (vii) exposure to a hypertonic medium by diluting in 1.5 M NaCl at a final concentration of 2 mg/mL.

Control samples were used throughout the study (samples of the medicine Opdivo^®^ 10 mg/mL not subjected to stress and analysed within the first 24 h after opening the medicine vial while ensuring no sample degradation). Both control samples and those subjected to stress were analysed at the concentration used in the medicine (10 mg/mL), in order to avoid alterations such as displacement of the aggregation equilibrium by dilution. In the CD study, the analysis required the dilution of the samples in reverse-osmosis-quality water (purified with a Milli-RO plus Milli-Q station from Merk Millipore, Darmstadt, Germany) at a concentration of 0.1 mg/mL to avoid detector saturation. The NaCl stressed sample and its corresponding control sample had to be analysed at 2 mg/mL since NaCl was added as a concentrated aqueous solution. All the stress tests were performed using 200 µL of the medicine (Opdivo^®^ 10 mg/mL), except for the high ionic strength test, for which a smaller volume of medicine was used. This was because a certain volume of NaCl had to be added to the sample at the right concentration to prepare a final volume of 200 µL. The temperature stresses were performed in 1.5 mL Eppendorf tubes (Eppendorf, Hamburg, Germany); the FTCs, agitation and high ionic strength tests were carried out in 2 mL amber RAM vials with a 9 mm thread (Symta, Madrid, Spain); and the light stress was performed in 2 mL clear RAM vials with a 9 mm thread (Symta, Madrid, Spain).

### 2.3. Physicochemical Analytical Methods

#### 2.3.1. Visual Inspections

A quick visual inspection was carried out each day, prior to experimentation, in order to check for evidence of formation of large aggregates, turbidity, suspended particles, colour changes, and gas formation. This inspection was conducted using the naked eye and carried out by three different analysts.

#### 2.3.2. Far Ultraviolet (UV) Circular Dichroism (CD) Spectroscopy

CD spectroscopy in the far region was used to study conformational changes in the nivolumab’s secondary structure over time. The experimental conditions were the same as those used in [18]. Opdivo^®^ spectra were recorded using a JASCO J-815 spectropolarimeter (JASCO, Tokyo, Japan) equipped with a Peltier system for temperature control. Temperature was set at 20 °C for all measurements. The concentration of the solution samples to be analysed was optimized to 0.1 mg/mL of nivolumab. Spectra were acquired from 250 to 190 nm every 0.2 nm with a scan speed of 20 nm/min. The CD spectrum of NaCl stressed samples was acquired from 250 to 200 nm due to the fact that the high salt concentration increased the voltage to over 700 V at a wavelength of less than 200 nm, and the voltage should be less than 700 V to obtain reliable data [19]. A total of five accumulations were averaged, with a bandwidth of 1 nm. A quartz cuvette with a path length of 1 mm was used. The first sample to be measured was the blank. The results for the blank were subtracted from all the samples so as to eliminate any possible interferences. Spectra Analysis software was used to apply the Means-Movement Smoothing to all the spectra. To determine the secondary structures, we used the Dichroweb website [20] and the most suitable algorithm and DataSet were CONTIN [21] and SP175 protein [22], respectively. A control sample was also subjected to a temperature ramp from 20 °C to 90 °C.

#### 2.3.3. Intrinsic Tryptophan Fluorescence Spectroscopy (IT-FS)

Conformational changes in the tertiary structure of nivolumab can take place even if the secondary structure remains unchanged. When the tertiary structure undergoes conformational changes, some hydrophobic pockets can be exposed to the solvent, which encourages the formation of aggregates. A Cary Eclipse spectrofluorometer (Agilent, Santa Clara, CA, USA) equipped with a Peltier system for temperature control was used to carry out IT-F measurements. The experimental conditions were similar to those used in [18]. Emission spectra were recorded from 300 to 400 nm and the excitation wavelength was set at 298 nm. The samples were analysed at 10 mg/mL and the temperature was set at 20 °C. Excitation and emission slits were set to 2.5 nm and 5 nm, respectively. A total of five spectral accumulations were recorded for all measurements and the scan speed was set at 600 nm/min. The spectral centre of mass (C.M.) was considered as a mathematical representation of each spectrum, and was calculated using the following equation:

(1) $$C.M.=\frac{\sum_{i}^{n}\left(\lambda \mathrm{ifi}\right)}{\sum_{i}^{n}\mathrm{fi}}$$

where λi is the wavelength and fi the fluorescence intensity.

#### 2.3.4. Dynamic Light Scattering (DLS)

A Zetasizer Nano-ZS90 Malvern (Malvern Instruments Ltd. Worcestershire, UK) was used to assess soluble particulates (from 1 to 10,000 nm) in Opdivo^®^ (10 mg/mL nivolumab) samples. The experimental conditions were similar to those used in [18]. A 1 cm spectrophotometry disposable cuvette was used to obtain the measurements. The temperature was set at 20 °C. A minimum of 100 reads were recorded per measurement and the acquisition time was 5 s per read. The average hydrodynamic diameter (HD), polydispersity index (PDI), polydispersity (Pd, %) and volume size distribution of all the samples were studied.

#### 2.3.5. Isoform Analysis by Size-Exclusion Ultra-High-Performance Liquid Chromatography (UHPLC) with UV-Visible Detection Coupled to (Native) Mass Spectrometry (SE/UHPLC-UV-[Native] MS) Using Volatile Salts

The analysis was performed by liquid chromatography using a Dionex UltiMate 3000 chromatograph (Thermo Scientific, Waltham, MA, USA), equipped with two ternary bombs; a degasser; an autosampler; a thermostatic column compartment; and a multiple-wavelength detector (MWD-3000 Vis-UV detector). The chromatograph was coupled in line to a Q Exactive™ Plus Hybrid Quadrupole Orbitrap mass spectrometer (Thermo Scientific). The ionisation was performed using a heated electrospray ionisation (HESI) source. Chromatographic and MS conditions were similar to those used in [23]. A SEC column 300 A, 2.7 μm, 4.6 × 300 mm (AdvanceBioSec, Agilent technologies, Santa Clara, CA, USA) was used and 8 µg of Opdivo^®^ was injected into the column. The column temperature was set at 30 °C. This analysis was carried out under isocratic conditions of 100 mM ammonium acetate buffer (LC-MS purity grade, Sigma Aldrich, Madrid, Spain) prepared in reverse-osmosis-quality water, at 0.3 mL/min for 20 min. The UV chromatograms were registered at different wavelengths, i.e., λ= 214 nm, λ= 220 nm and λ = 280 nm, using a bandwidth of 5 nm in all cases. The MS method was carried out in full positive polarity mode at a resolution of 17,500 (defined at *m/z* 200). The mass range was set at 2500–6000 *m*/*z* and the automatic gain control (AGC) target value was 3.0 × 10^6^ with a maximum injection time of 200 ms and 10 microscans. In-source collision-induced dissociation (CID) was set to 100 eV. The MS instrumental parameters were set as follows: the sheath gas flow rate was 20 arbitrary units (AU); auxiliary gas flow rate was 5 AU; spray voltage was 3.6 kV; capillary temperature was 275 °C; probe heater temperature was 275 °C; and S-lens RF voltage was 100 V.

The analysis was performed by liquid chromatography using a Dionex UltiMate 3000 chromatograph (Thermo Scientific, Waltham, MA, USA), equipped with two ternary bombs; a degasser; an autosampler; a thermostatic column compartment; and a multiple-wavelength detector (MWD-3000 Vis-UV detector). The chromatograph was coupled in line to a Q Exactive™ Plus Hybrid Quadrupole Orbitrap mass spectrometer (Thermo Scientific). The ionisation was performed using a heated electrospray ionisation (HESI) source. Chromatographic and MS conditions were similar to those used in [23]. A SEC column 300 A, 2.7μm, 4.6 × 300mm (AdvanceBioSec, Agilent technologies, Santa Clara, CA, USA) was used and 8 µg of Opdivo^®^ was injected into the column. The column temperature was set at 30 °C. This analysis was carried out under isocratic conditions of 100 mM ammonium acetate buffer (LC-MS purity grade, Sigma Aldrich, Madrid, Spain) prepared in reverse-osmosis-quality water, at 0.3 mL/min for 20 min. The UV chromatograms were registered at different wavelengths, i.e., λ= 214 nm, λ= 220 nm and λ = 280 nm, using a bandwidth of 5 nm in all cases. The MS method was carried out in full positive polarity mode at a resolution of 17,500 (defined at *m/z* 200). The mass range was set at 2500–6000 *m*/*z* and the automatic gain control (AGC) target value was 3.0 × 10^6^ with a maximum injection time of 200 ms and 10 microscans. In-source collision-induced dissociation (CID) was set to 100 eV. The MS instrumental parameters were set as follows: the sheath gas flow rate was 20 arbitrary units (AU); auxiliary gas flow rate was 5 AU; spray voltage was 3.6 kV; capillary temperature was 275 °C; probe heater temperature was 275 °C; and S-lens RF voltage was 100 V.

Native isoforms of the medicine and stressed samples were obtained by mass spectra deconvolution using BioPharma Finder™ software version 3.1 (Thermo Scientific) with ReSpect algorithms.

#### 2.3.6. Aggregation Study by Size-Exclusion Ultra-High-Performance Liquid Chromatography (UHPLC) with UV-Visible Detection (SE/UHPLC-UV) Using Non-Volatile Salts

The aggregation study was conducted by liquid chromatography using the chromatograph (Dionex UltiMate 3000 chromatograph, Thermo Scientific) and SEC column (300 A, 2.7 μm, 4.6 × 300mm, AdvanceBioSec) described above. In this particular analysis, the chromatograph was used separately from the Q Exactive™ Plus Hybrid Quadrupole Orbitrap mass spectrometer. Therefore, the mobile phase was composed of non-volatile salts: 150 mM of phosphate buffer pH 7.0, which was prepared with monohydrate monobasic sodium phosphate (Panreac, Barcelona, Spain) and anhydrous disodium hydrogen phosphate (Panreac, Barcelona, Spain) in reverse-osmosis-quality water. The analysis was performed under isocratic conditions for 18 min. The column temperature was set at 30 °C and 8 µg of sample (Opdivo^®^) were injected into the column at 0.3 mL/min. The UV chromatograms were registered at 214 nm, 220 nm and 280 nm, using a bandwidth of 5 nm in all cases.

The SEC column was calibrated to establish the relationship between the molecular weight and the retention time of nivolumab. The calibration kit (Agilent, Santa Clara, CA, USA) was composed of five proteins: thyroglobulin (670 kDa), γ-globulin (150 kDa), ovalbumin (45 kDa), myoglobin (17 kDa) and angiotensin II (1 kDa), although this last protein was not used in the calibration of the SEC column, due to its low molecular weight (M.W.). The experimental size exclusion column calibration model is M.W. = 1 × 10^8^e^−0.828x^; R^2^ = 0.9989.

### 2.4. Functional-Based Method: Enzyme-Linked Immunosorbent Assay (ELISA)

In order to test the biological activity of nivolumab, we developed and optimised a new indirect, non-competitive ELISA method on the basis of the available bibliography [24,25,26] and of our previous research into other therapeutic mAbs [18,27,28]. The optimised method was as follows: firstly, 96-well Maxisorp immune plates were sensitised by adding 100 μL/well of 0.5 μg/mL Human PD-1 (CD279), FC Fusion (Sigma Aldrich, Madrid, Spain) diluted in 0.1 M carbonate buffer solution pH 9.6 (prepared with sodium carbonate (Panreac, Spain) and sodium bicarbonate (Panreac, Spain)) and incubated overnight (18 h) at 4 °C. The plates were manually washed four times with 200 μL/well of PBS-Tween 20 pH 7.4 containing 0.3% (*v*/*v*) Tween 20^®^. PBS was prepared using sodium chloride, potassium chloride, disodium phosphate monohydrate and potassium phosphate monobasic supplied by Panreac (Barcelona, Spain), while Tween 20 was supplied by Guinama (Valencia, Spain). The plates were then treated with 200 μL/well of the blocking buffer (PBS-Tween 20 pH 7.4 containing skimmed milk 2% (*w*/*v*)) for 2 h at 37 °C to eliminate nonspecific absorptions. After that they were washed four more times and filled with a 100 μL/well of nivolumab appropriately diluted in 0.1 M carbonate buffer (pH 9.6) at three concentrations: 5 ng/mL, 10 ng/mL and 25 ng/mL. The plates were incubated at 37 °C for 45 min in a universal digitronic precision oven P Selecta^®^ (J.P. Selecta, s.a. Abrera, Barcelona, Spain), after which they were washed four times with PBS-Tween 20 and incubated again with 100 μL/well of 1:1000 in PBS diluted anti-human IgG4-HRP (mouse anti-human IgG4 Fc antibody-HRP conjugate, Thermo Fisher, Landsmeer, the Netherlands) for 30 min at 37 °C. After being washed for four times, 100 μL/well of the substrate solution (O-Phenylenediamine Dihydrochloride (OPD), Sigma Aldrich, Madrid, Spain) were added and incubated for 20 min at room temperature (around 25 °C) in darkness. Finally, 50 μL/well of 1M sulfuric acid solution was added to stop the reaction. Absorbance was recorded at 450 nm and 620 nm, and the analytical signal was the difference between the two absorbance values (NanoQuant Infinite 200 Pro, Tecan, Austria, GMBH).

This ELISA format allowed us to assess the specific interaction between nivolumab and its target, the PD-1 receptor. In this way, we were able to evaluate and quantify the biological activity of nivolumab after applying the stress factors indicated previously. To this end, the method was validated in terms of the calibration model, precision and accuracy.

The next stage was to investigate the standard calibration model using standard samples of nivolumab. In this case, fresh samples of the medicine Opdivo^®^ (10 mg/mL nivolumab) were used as the standard due to the lack of any proper standard samples of nivolumab. The nivolumab concentrations checked to obtain the calibration model were 100, 50, 25, 10, 5, 1, and 0.5 ng/mL diluted in 0.1 M carbonate buffer (pH 9.6). Each standard concentration was prepared and analysed in quintuplicate. From the graphical distribution results (absorbance vs. ng/mL nivolumab), the selected optimum concentrations for analysing nivolumab were 25, 10 and 5 ng/mL, as the corresponding response values were widely distributed within the permitted absorbance range, i.e., from 0 to 1. The Statgraphics Centurion 18 (Statgraphics Technologies, The Plains, VA, USA) software package was used for the statistical analysis of the calibration models. The coefficient of determination (R^2^) and the lack-of-fit signification test were used to evaluate the best fit of these data to the different mathematical models. Once these concentrations were selected as representative of the calibration model, the precision and the accuracy of this ELISA method were evaluated according to the ICH recommendations Q2(R1) at these three concentrations [29]. The precision was checked as repeatability (intraday precision) and intermediate precision (interday precision; over three consecutive days). For the repeatability study, nine standard solutions were prepared within the same day and under the same experimental conditions: 3 samples at 5 ng/mL, 3 samples at 10 ng/mL and 3 samples at 25 ng/mL. Intermediate precision was assessed by analysing three standard solutions (5, 10 and 25 ng/mL of nivolumab) that were freshly prepared on three different days and under the same experimental conditions. Both repeatability and intermediate precision were determined as the relative standard deviation (RSD, %) of the concentrations calculated from the absorbance using the standard calibration curve. Accuracy was assessed by analysing three standard solutions of each concentration of nivolumab and determining the mean recovery percentage (R, %) for each concentration.

The functional study of the stressed samples was performed by simultaneously analysing control (fresh) and stressed nivolumab samples (both from Opdivo^®^) using the validated ELISA method. The results were then statistically compared (Student’s *t* analysis with 95 % of confidence) to find out whether the ability of nivolumab to specifically bind to its target, the PD-1, had been altered in any way. To this end, the biological activity of each of the stressed samples was compared with that of the control sample. The reported biological activity was the average of three replicates.

## 3. Results

### 3.1. Visual Inspections

All the samples remained clear after the different stress conditions considered in this study had been applied. This means that no precipitates or particulate matter could be detected with the naked eye, even in the samples exposed to 60 °C for one hour.

### 3.2. Far Ultraviolet (UV) Circular Dichroism (CD) Spectroscopy

**Figure 1:**
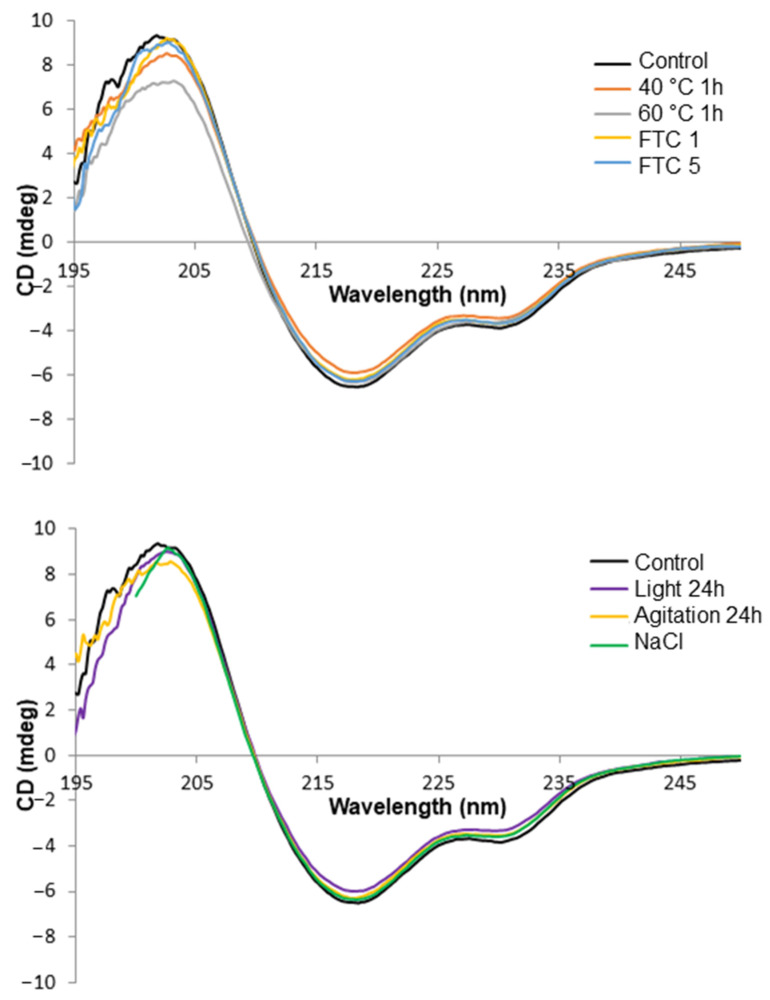
Far-UV CD spectra of all the stressed samples of nivolumab as compared with a control sample (black line).

The characteristic Far UV spectra of the nivolumab control and stressed samples are shown in Figure 1. The CD spectrum of the control sample was characterised by the wavelength of 209.7 ± 0.1 nm with ellipticity = 0, by the wavelength of the minimum at 218.0 ± 0.4 nm and by a shoulder at 228.7 ± 0.1 nm. As regards the stressed samples, the three aforementioned checkpoints were monitored in order to detect any changes in the secondary structures of nivolumab. The results obtained (Table 1) show that the characteristic wavelengths in the stressed samples remained unchanged with respect to the control sample, so indicating that the various stress tests we performed did not cause any changes in the secondary structure of nivolumab.

As expected, the secondary structure content showed a majority of β strands and random coil conformation after mathematical deconvolution of the CD spectra (Table 2). There were no significant changes when the stressed samples were compared to the control sample, as might be expected given the results of the CD spectra analysis. We were unable to assess the secondary structure content of the sample exposed to high ionic strength, because the high NaCl content of the solution meant that we had to record the spectra from 200 nm to 250 nm, an insufficient wavelength range for analysis by the selected algorithm and the protein dataset.

We also carried out a temperature ramp to check the thermal stability of nivolumab (0.1 mg/mL). The selected temperature range was from 20 °C (ambient conditions) to 90 °C (high stress conditions). The results indicated that the CD spectra remained unaltered up to 60 °C (Figure S1), so maintaining the values of the characteristic CD spectral parameters indicated above. By contrast, temperatures of over 60 °C caused significant spectral changes. On the basis of the results of the temperature ramp, we were able to select the most appropriate temperatures to carry out the thermal stress test. We therefore decided to subject nivolumab to 40 °C, an easily reachable temperature in the summer in Spain, and 60 °C, the maximum temperature at which nivolumab remains unaltered, at least in terms of its secondary structure.

### 3.3. IT-FS

**Figure 2:**
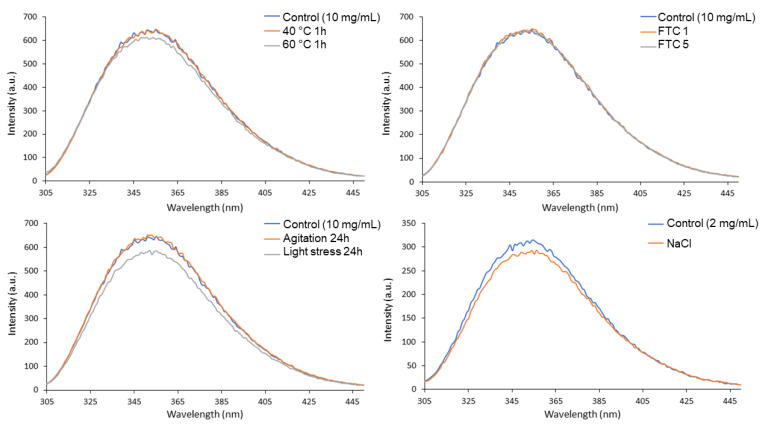
Nivolumab stress studies by IT-FS.

Figure 2 shows the fluorescence spectra for the control and stressed samples of nivolumab. Most of the spectra are very similar in terms of both shape and intensity. The only spectrum to show different intensity was the one obtained after exposing the nivolumab sample to light, which showed decreasing intensity due to a decrease in the fluorescence signal of the tryptophan residues of nivolumab. This is probably due to degradation, as light stress is a well-known oxidising agent [30]. Tryptophan residues may be oxidised, so decreasing the intensity of the IT-FS spectrum. This degradation could lead to differences in long-term stability, bioactivity and immunogenicity [31].

The spectra were used to calculate the C.M. value, a number used to characterise each sample in order to be able to detect any slight modifications in the tertiary structure of nivolumab more effectively. Slight modifications in the C.M. value with respect to the control samples indicate conformational changes in the spatial arrangement of the tryptophan residues; a shift to lower values indicates that the tryptophan residues are buried deep within the core of the protein, which has a more hydrophobic environment, while a shift to higher C.M. values indicates that the tryptophan residues are more exposed to the solvent, and therefore that the structure of the protein has unfolded to some extent [32]. Table 3 shows the C.M. values for the fluorescence spectra of control and stressed samples. No modifications were detected in the C.M values of the stressed samples as compared to the control. This indicates that the stress conditions tested using this technique did not cause any conformational changes in the tertiary structure of the protein.

### 3.4. DLS

**Figure 3:**
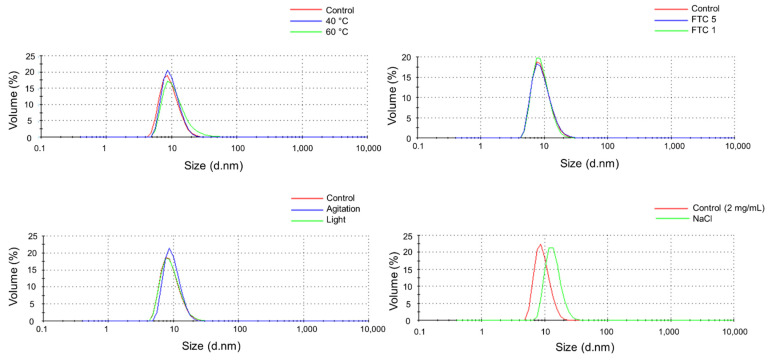
Particulate size distributions by volume in control and stressed samples of nivolumab.

DLS results are shown in Table 4. Particulate size distributions by volume in the nivolumab (Opdivo^®^, 10 mg/mL) medicine and stressed samples are shown in Figure 3. Control samples (Opdivo^®^, 10 mg/mL nivolumab) showed a single population of particles attributed to the monomers of nivolumab with a hydrodynamic diameter (HD) of 9.7 ± 3.2 nm and 9.3 ± 3.2 nm for control 1 and control 2, respectively (two control samples were considered as the analyses were performed on different days). All the stressed samples remained unaltered, with no new particulate population detected up to 10 μm diameter size. All the samples had monomer populations of similar size, except for the one exposed to high temperature (60 °C, 1 h), which showed a slight increase in the HD. This could be attributed to increased hydration of the nivolumab monomers at this temperature, which was close to the temperature at which the secondary structure of nivolumab begins to degrade (as demonstrated above in the temperature ramp).

The dilution required to subject nivolumab (Opdivo^®^, 10 mg/mL) to the stress of NaCl media (2 mg/mL) did not affect the single monomers’ population detected in the medicine. The control sample prepared by diluting the medicine with water showed a single population with a HD of 9.4 ± 2.6 nm, which indicates that the HD was unaffected. In the nivolumab sample stressed with NaCl the HD increased to 13.9 ± 3.8 nm, which indicates that nivolumab may be slightly affected by exposure to a high ionic strength medium, and the electrostatic interaction between the surface of nivolumab and the electrolyte ions expands the volume of the protein. This may be due to an increase in hydration on the surface of the protein, a result that coincides with the findings of previous research on mAbs [18].

We also assessed the polydispersity index (PDI) for each sample in order to corroborate that there was just one particulate population (i.e., monomers) in all the samples of nivolumab. PDI is a dimensionless parameter, which is scaled from 0 to 1, but is almost always greater than 0.05, which indicates highly monodisperse standards. It is generally accepted that a sample is highly monodisperse when the PDI value is ≤0.1 and moderately polydisperse when its value is between 0.1 and 0.4. Values above 0.7 indicate that the sample is probably not suitable for studying with the DLS technique as it has a very broad size distribution. As expected, given the standard deviation (SD) values for the HD, the PDI was around 0.1 and 0.2 for the majority of the samples, which confirmed that the solutions were moderately polydisperse. The samples exposed to 60 °C and NaCl showed a PDI of 0.32 and 0.35, respectively. These values indicate a higher degree of polydispersity, as also indicated by the SD values (5.8 and 3.8, respectively) of the HD in these samples.

The polydispersity (%, *Pd*) of the single population was calculated for each sample using the following equation:

(2) $$Pd=\frac{{\left(St.\;Dev\right)}^{2}}{{\left(Size\right)}^{2}}*100$$

where *St.Dev* is the SD (nm) of the population and *Size* (nm) is the mean size of the population. The *Pd* (%) measures the width of the assumed distribution. In terms of protein analysis, a *Pd* ≤ 20% indicates that the sample is monodisperse. In the DLS study, the results obtained by intensity were used to calculate the *Pd* (%) of each nivolumab sample (Table 4). All samples showed a polydispersity of less than 20%, except for the one subjected to the 60 °C stress test (60.7%). This indicates that this sample is polydisperse, as might be expected from the SD of the monomer population.

### 3.5. Isoform Analysis by SE/UHPLC(UV)-[Native]MS

**Figure 4:**
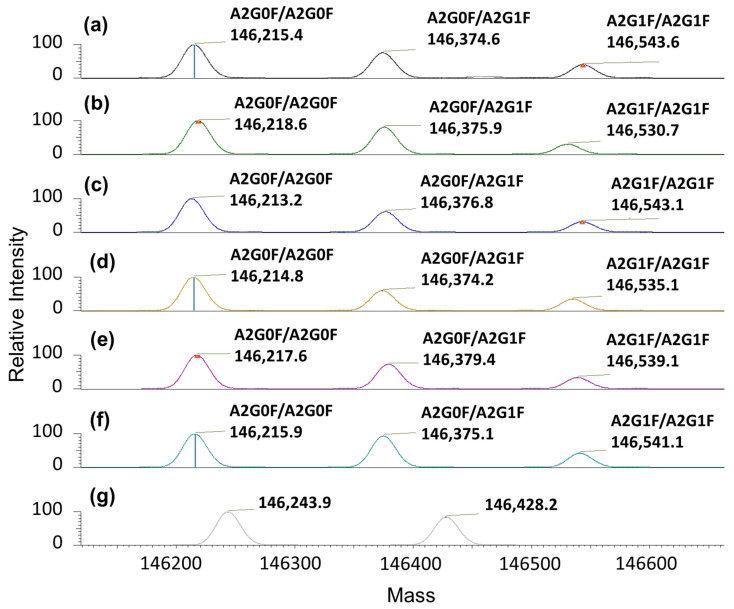
Deconvoluted mass spectra for nivolumab samples (10 mg/mL) showing the mass isoform profile with their proposed N-glycoforms: (**a**) fresh/control sample; (**b**) 40 °C/1 h exposure stress; (**c**) 60 °C/1 h exposure stress; (**d**) FTC 1; (**e**) FTC 5; (**f**) agitation stress; and (**g**) light stress.

The LC-MS method was used to identify the particular isoform profile of fresh and stressed nivolumab samples [23]. The use of volatile salts in the mobile phase (low ionic strength) was essential to enable the chromatographic system to be coupled directly to the mass detector. The nivolumab mass spectra profile was characterised under native conditions for all the samples. Nivolumab control samples showed three main isoforms, i.e., 146,215.4 (the most intense), 146,374.6 and 146,543.6 Da (the least intense). The first was assigned to the A2G0F/A2G0F glycoform, the second was assigned to the A2G0F/A2G1F and the third was assigned to the A2G1F/A2G1F (Figure 4a). These principal glycoforms were also identified in a parallel study of intact nivolumab under denatured conditions (manuscript in preparation). This means that the main nivolumab isoforms have now been identified under both native and denatured LC-MS, so giving additional support to these findings.

Most of the isoform profiles for the stressed samples remained unchanged when compared to the control sample (Figure 4). The three main isoforms proposed for nivolumab in the control sample were exactly the same as those proposed in the stressed samples, so indicating that there were no significant chemical modifications in the main isoforms (Table 5). The only exception were the samples subjected to accelerated light degradation. In this case, in addition to the reduction in the number of isoforms detected, these isoforms had different masses (see Figure 4g), so indicating degradation of the protein. It is important to note that no isoform profile was obtained for the NaCl stress test. This was because the mass detector was unable to analyse the protein due to the excessive amount of salt used in this test. The N-glycan profiles for the control and stressed samples of nivolumab are presented in Table 5.

### 3.6. Aggregation Study by SE/UHPLC(UV)

Aggregation is one of the main indicators of protein degradation. In order to obtain the aggregates’ profile of nivolumab, fresh and stressed samples were analysed by SE/UHPLC(UV) and their chromatographic profiles were compared in order to detect changes. The SE chromatogram for the control sample of nivolumab (fresh medicine 10 mg/mL) and the experimental size exclusion column calibration model are shown in Figures S2 and S3, respectively. In the SEC aggregation profile for the control sample, an important peak can be observed at 8.70 ± 0.01 min (99.6 ± 0.06% of the total area), which was assigned to nivolumab monomers. Just before the main peak there is a small peak eluting at 7.84 ± 0.02 min (0.4 ± 0.06% of the total area), which was assigned to the natural dimers present in the medicine. Assignment of the peaks was based on the indications of the specific chromatographic column and on the calibration of the column, performed using a commercial protein standard kit.

**Figure 5:**
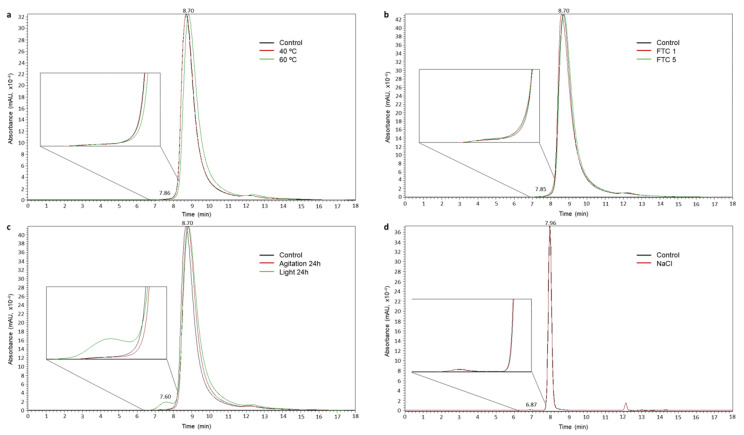
SE/UHPLC(UV) chromatograms for 10 mg/mL samples of nivolumab: (**a**) temperature stress; (**b**) FTC stress; (**c**) agitation and light stresses; and (**d**) ionic strength stress.

As regards the forced degradation study, Figure 5 shows the SE chromatograms resulting from the different stresses to which the nivolumab samples were exposed. The retention times and proportions of the peaks detected in their chromatographic profiles are presented in Table 6. No changes were detected in the SEC profile of the stressed samples of nivolumab and the relative proportions of monomers and dimers remained constant, except in the samples exposed to light (24 h) in which the dimers’ peak was proportionally bigger. It was estimated that exposure to light stress caused 3% of the monomers to be transformed into dimers in 24 h (Table 6).

During the course of the aggregation study, we had to replace the SEC column we were using with a new one. This change resulted in a modification of the chromatographic profiles as the new column showed higher peak resolution capability. In the chromatogram in Figure 5d, the chromatographic peaks for the control and NaCl stressed samples are thinner and the retention times are shorter than the chromatograms in the other panels (a, b and c), because the NaCl stress study was carried out using the new column.

A non-assigned chromatographic peak was detected in all the SE chromatographic profiles at a retention time of 12.20 ± 0.02 min. However, this peak is not a sign of degradation as it was also detected in the control (fresh) medicine samples.

### 3.7. ELISA

The biological activity of nivolumab (antibody-antigen binding specificity) can be evaluated by ELISA-based binding assays. When exposed to stress, mAbs can undergo physicochemical modifications that may impair their effectiveness. The ELISA technique can be used to evaluate the impact of these modifications [27,28].

We developed a new ELISA method for this study. The first stage was therefore to validate the ELISA. The calibration model was established by selecting the mathematical function that best fits the experimental data (standard calibration curve in Figure S4). After reviewing various different mathematical models, a quadratic polynomial model was selected as the most suitable for calibration purposes, given its R^2^ (99.34%) and *p*-value (0.0252), which demonstrate, respectively, the goodness of fit and the significance of the selected mathematical model (Table 7).

Other important aspects of validation include repeatability and intermediate precision, both expressed in terms of their RSD (%), as set out in Table 8. Accuracy, expressed as R (%), is also presented in this table. For immunoassays, minimal acceptance limits of 20% are recommended, which means that the model fulfilled the precision criteria indicated for bioanalytical method validation [33,34]. The recovery values were between 92% and 106%, so demonstrating the high quality of the ELISA method we proposed for nivolumab, especially considering the huge variability inherent in immunoassay-based methods [28].

**Figure 6:**
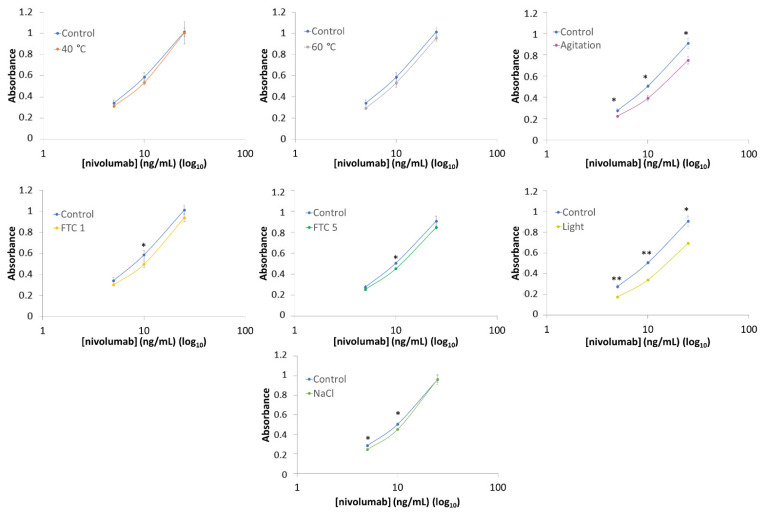
ELISA binding assay (5, 10 and 25 ng/mL) for the stressed samples as compared to a fresh (control) sample of nivolumab (*: *p*-value < 0.05; **: *p*-value < 0.001).

Once the ELISA had been fully validated, it was used to assess the biological activity of the stressed nivolumab samples. Figure 6 shows the results of the ELISA assays. Each stressed sample was analysed by obtaining the calibration graph, which was compared with the graph obtained in the analysis of the control (fresh) samples. In order to detect statistically significant differences, a Student’s *t* analysis was carried out as a means of comparing the experimental data from control and stressed samples: in Figure 6 the results marked with asterisks are significantly different (one asterisk, *p*-value < 0.05) and very significantly different (two asterisks, *p*-value < 0.001) from the control. The results show that the samples of nivolumab subjected to heat (40 °C/1 h and 60 °C/1 h) retain their functionality as the control sample. The samples subjected to stress by agitation and light exposure showed a clear loss in PD-1 binding capacity, as revealed by the shift towards lower absorbance values of the graphical function for the stressed samples as compared to the graphical function for the control samples; a remaining PD-1 binding capacity of 80% (agitation) and 70% (light stress exposure) was estimated, after assuming a binding capacity of 100% in the control samples in each graph (Table 9). In the case of the samples subjected to FTC 1 and 5, results indicated a slight decrease in PD-1 binding capacity to PD-1 with respect of the control samples, estimating the remaining biological activity at around 90% for both samples. This is in line with the instruction in the Opdivo^®^ technical reports about not freezing the medicine [4]. The ELISA assays also revealed two significant values (Figure 6) in the sample subjected to high ionic strength stress (NaCl). Therefore, the biological activity of the stressed sample differs from the control samples, with a remaining activity of around 90% (Table 9).

## 4. Discussion

Throughout this study, we used samples of nivolumab in its medicine formulation (Opdivo^®^, 10 mg/mL). We began by characterising fresh control samples of nivolumab, which were then compared with stressed samples that had been subjected to a range of different stress conditions. The aim of these tests was to detect and identify possible changes in the medicine such as aggregation, denaturation or structural modifications, and the appearance of new particulates.

Results indicate that nivolumab medicine (Opdivo^®^, 10 mg/mL) is composed of monomers and natural dimers in relative proportions of 99.6% and 0.4%, respectively, as highlighted in the SEC profiles conducted on the batches (Table 6). The native LC-MS(Orbitrap) isoform profile indicated three main glycoforms, which were assigned to A2G0F/A2G0F, A2G0F/A2G1F and A2G1F/A2G1F, in accordance with their particular masses (Figure 4 and Table 5). As regards the secondary structure of nivolumab derived from the CD spectrum, this consisted mainly of β-sheet (Table 2), as is normal in mAbs [18,27], and showed the characteristic spectral parameters: minimum at around 218.0 nm, shoulder at 228.7 nm and wavelength for ellipticity = 0 of around 209.7 nm. The tertiary structure, which was characterised by IT-FS, showed a centre of spectral mass (C.M.) of 361 nm (Table 3) for both the undiluted (10 mg/mL) and diluted (2 mg/mL) medicine. The DLS study indicated an HD of around 9.7 nm and 9.3 nm and a polydispersity of 11.7% and 12.4% for the undiluted nivolumab samples used as control, while the diluted medicine showed similar HD (9.4 nm) and polydispersity (7.4%) (Table 4). All these values are shared with other therapeutic mAbs [18,27].

The heat stress study was carried out by subjecting the medicine (Opdivo^®^, 10 mg/mL) to two different temperatures, 40 °C and 60 °C, for 1 h. In both cases, there were no significant changes in the medicine. SE/UHPLC(UV) analysis did not show any increase in the dimer population or the appearance of high molecular weight (HMW) aggregates (Figure 5). The lack of aggregation was also corroborated by DLS, which only detected one population of particulate, i.e., the monomers. However, in the sample subjected to 60 °C, there was an increase in HD (from 9.7 ± 3.2 nm in the control to 11.7 ± 5.8 nm in the stressed sample) and PDI (from 0.21 in the control to 0.32 in the stressed sample), which could be attributed to an increased hydration of the nivolumab monomers; this could be due to the fact that the heat stress temperature (60 °C) was very close to the temperature beyond which the secondary structure began to deteriorate (65 °C) (Table 4). In addition, CD and IT-FS did not detect any changes in the secondary and tertiary structures of the protein (Table 1 and Table 2). For its part, the isoform profile was very stable and the glycoforms remained unaltered. The same glycoforms, with similar relative abundances, were identified in both the heat-stressed and control samples of nivolumab (Figure 4 and Table 5). Moreover, ELISA assays demonstrated that subjecting nivolumab to 40 °C and 60 °C for 1 h did not affect its functionality, as measured in terms of its capacity to bind to its target (the PD-1) (Figure 6). Although previous researchers reported slight structural changes in IgG4 [35] and in nivolumab [7] when subjected to more aggressive heat stress, in this study we have demonstrated that nivolumab (Opdivo^®^ 10 mg/mL) remains stable when subjected up to 60 °C for 1 h, and its binding capacity to PD-1 is unaffected.

It is well known that freeze/thaw cycles (FTC) can lead to protein aggregation, as they disturb the local structure on the surface of the residues of the protein, so giving rise to partial denaturation during freezing, which culminates in an aggregation process [36]. Nevertheless, no changes were noted in nivolumab in either FTC 1 or FTC 5. In both cases, the secondary and tertiary structures remained stable, as did the relative proportions of monomers and dimers. Moreover, DLS confirmed the absence of aggregates and the isoform profile remained unaltered. ELISA results showed that the nivolumab-PD-1 binding capacity decreased slightly to around 90% after one and five cycles. This means that this functional property is slightly affected by the freeze/thaw cycles, even though none of the physicochemical parameters that we studied indicated modifications in the medicine (Figure 6). As indicated earlier, this result is in line with the instruction in the technical report on Opdivo^®^ [4] about not freezing this medicine.

Agitation for 24 h had no significant effects on the particulate in the nivolumab sample. SE/UHPLC(UV) demonstrated that there were no changes in the relative proportions of monomers and dimers, and DLS also confirmed that no new populations or aggregates appeared after application of this stress. In addition, the CD results showed a similar spectrum to the control sample, so indicating that the secondary structure remained unchanged. The same occurred with the tertiary structure, given that the IT-FS results for the stressed sample had a similar profile to the control, and their C.M. values were exactly the same. The isoform profile also remained unchanged, as revealed by the fact that the glycoforms assigned to the stressed sample matched those assigned to the control one. However, ELISA indicated a reduction in the capacity of nivolumab to bind to PD-1 (Figure 6 and Table 9), which could not be due to any structural modification, given the aforementioned results. However, it could be due to changes in the tertiary structure of nivolumab, given that the maximum emission of tryptophans in nivolumab is over 350 nm, which is a very high value, similar to that of the amino acid in aqueous solution [37]. This indicates that the tryptophans in nivolumab are close to a hydrophilic environment, and are therefore highly exposed to the solvent. As a result, IT-FS is unable to detect the structural changes that might lead to the loss of biological activity revealed by ELISA. In the near future, we will be investigating these findings in much greater detail using peptide mapping analysis.

As regards the stress involving exposure to light for 24 h, non-natural aggregation was observed by SE/UHPLC(UV) (Figure 5). Previous research on other mAbs has shown that exposure to light induces aggregation [30,38], a finding that was also true for nivolumab medicine samples, in which the SE-chromatographic profile showed an increase in the peak for dimers, whose relative proportion was 3% higher than in the control sample (Table 6). Despite the increased proportion of dimers, CD assessment did not detect any changes in the secondary structure of nivolumab. As regards the tertiary structure of the protein, changes were detected, not in the C.M. (indicating no protein unfolding) of the IT-FS spectra, but in their intensity. This was probably due to the light-triggered oxidation of tryptophan residues. These changes may have caused the loss of biological activity, which could also have been induced by the aggregation of mAbs, as reported in previous research [39]. As expected, the nivolumab sample exposed to light showed a statistically highly significant decrease in the binding capacity of nivolumab to PD-1 (Figure 6), falling to around 70% of that of the control sample (Table 9). The isoforms profile was also very different because of the chemical changes (i.e., oxidations) caused by light exposure, which modified the molecular weight of nivolumab. This shows that the isoform profile was greatly affected by exposure to light despite being unaffected by the other forms of stress (Figure 4). Two new nivolumab isoforms were detected after the light exposure test with different masses compared to those detected in the control sample. No N-glycoforms could be attributed to these isoforms. All these results clearly indicated that exposure to light stress caused important degradation of nivolumab. These changes were probably due to the oxidative process to which the sample is exposed, as many previous publications suggest. We obtained similar results in a parallel study of nivolumab (manuscript in preparation).

As regards the stress by exposure to high ionic strength, the SE/UHPLC(UV) and DLS results indicated that no aggregation occurred, as monomers and dimers appeared in the same proportions as in the control sample and no HMW aggregates were detected, either by SEC or by DLS. Nevertheless, DLS results showed an increase in the HD value (from 9.4 ± 2.6 nm in the control to 13.9 ± 3.8 nm in the stressed sample) and in the PDI (from 0.29 in the control to 0.34 in the stressed sample). This was attributed to an increase in hydration, as indicated in Section 3.4 DLS (Table 4). CD results indicated that the secondary structure of nivolumab was not affected by this stress (Table 3). Despite all these results, ELISA indicated two significant values in Figure 6, referring that the binding capacity of nivolumab to the PD1 slightly (and significantly) decreased (around a 10%) with respect to the control samples. This could be due to the proposed increase in the hydration of the surface of the protein due to the increase in ionic strength, which might affect the capacity of nivolumab to bind to PD-1, its therapeutic target. The changes detected by DLS may therefore contribute to this loss of biological activity.

## 5. Conclusions

Biopharmaceuticals can be exposed to a wide range of environmental stress conditions during routine handling in hospitals, as well as in previous stages such as research, development and manufacturing. The research findings presented in this paper focus above stresses to which nivolumab (Opdivo^®^) might be subject when handled in hospital prior to administration. To assess the impact of these stresses, various stress tests were performed. The results of these tests indicate that nivolumab is most affected by accelerated exposure to light. This is because light causes non-natural aggregation (dimerisation) of the protein and promotes oxidations (i.e., tryptophan residues). As a consequence of these chemical changes, it also alters the isoform profile. The results of the functional studies, which measured the capacity of nivolumab to bind to the PD-1 receptor, demonstrated that this capacity is substantially reduced when nivolumab is subject to stress by exposure to light. This means that it should be stored and handled away from the light so as to avoid degradation. On the other hand, nivolumab (Opdivo^®^) was found to remain highly stable when subjected to heat exposure for 1 h up to 60 °C, even though this was very close to the temperature at which nivolumab starts to deteriorate due to heat stress. As regards the agitation test, even though no significant conformational changes, aggregation or particulate were observed, the biological activity of nivolumab was affected, in that it caused a decrease in the ability of nivolumab to bind to the PD-1 receptor (estimated at around 20%). We therefore strongly recommend, as far as possible, to avoid shaking the medicine and its pharmaceutical preparations and to take great care during transport and handling. Caution must also be taken when diluting the medicine in NaCl, as physical modifications were detected when nivolumab was exposed to a highly hypertonic solution. We have also confirmed that freezing/thawing could affect the capacity of nivolumab to bind to PD-1, a finding that confirms the instruction in the Opdivo^®^ technical report about not freezing the medicine. As expected, this research highlights the fragile nature of biopharmaceutics and, in particular, of nivolumab.

## Figures and Tables

**Table 1:** Secondary structures of nivolumab samples tracked by changes in the Far UV CD spectra.

Stress	Wavelength (nm) (Ellipticity = 0)	Minimum (nm)	Broad Shoulder (nm)
Control	209.7 ± 0.1	218.0 ± 0.4	228.7 ± 0.1
40 °C 1 h	210	218.2	228.8
60 °C 1 h	209.4	218.2	228.6
FTC 1	209.8	217.8	228.8
FTC 5	209.9	218.2	228.4
Agitation 24 h	209.8	217.8	228.4
Light stress 24 h	209.8	218.2	228.8
NaCl	209.6	218.2	228.4

**Table 2:** Percentage estimation of the different secondary structures by Dichroweb from the Far UV CD spectra.

Stress	Helix 1	Helix 2	Strand 1	Strand 2	Turns	Unordered
Control	0	4.8	30.8	14.1	10.1	40.1
40 ºC 1 h	0.1	4.8	31.6	14.2	10.2	39.1
60 ºC 1 h	0	4.9	31.1	14.5	10.4	39.2
FTC 1	0	4.3	31.3	13.9	10.8	39.8
FTC 5	0.1	4.7	32.5	14.6	10.2	37.9
Agitation 24 h	0	4.7	32.5	14.7	9.9	38.1
Light stress 24 h	0.1	4.8	32.8	14.9	9.9	37.5
NaCl	-	-	-	-	-	-

**Table 3:** Nivolumab tertiary structure assessed using the C.M. of the IT-FS spectrum.

Sample	Stress	Centre of Spectral Mass (C.M.) (Fluorescence Spectrum)
10 mg/mL	Control	361
	40 °C 1 h	361
	60 °C 1 h	361
	FTC 1	361
	FTC 5	361
	Agitation 24 h	361
	Light stress 24 h	361
2 mg/mL	Control	361
	NaCl	361

**Table 4:** Average hydrodynamic diameter (HD), polydispersity index (PDI) and polydispersity (%, Pd) of nivolumab sample solutions. Results that are different from the control appear in **bold**.

Sample	Stress	HD (nm)	PDI	Pd (%)
10 mg/mL	Control 1	9.7 ± 3.2	0.21	11.7
	40 °C 1 h	10.0 ± 3.1	0.22	9.5
	60 °C 1 h	**11.7 ± 5.8**	**0.32**	**60.7**
10 mg/mL	Control 2	9.3 ± 3.2	0.13	12.4
	FTC 1	9.1 ± 2.8	0.09	9.4
	FTC 5	9.4 ± 3.4	0.15	14.4
	Agitation 24 h	9.8 ± 2.8	0.07	7.8
	Light stress 24 h	9.4 ± 3.3	0.13	13.3
2 mg/mL	Control	9.4 ± 2.6	0.29	7.4
	NaCl	**13.9 ± 3.8**	**0.35**	6.9

**Table 5:** Experimental masses for intact nivolumab in native conditions for control and stressed samples. The relative abundances of N-glycoforms are also reported. Theoretical masses were calculated considering two C-term lysine clipping, two N-term pyroglutamate formation and 16 disulphide bonds.

	Glycoforms Associated	Experimental Mass (Da)	Theoretical Average Mass (Da)	Mass Difference (Da)	Mass Difference (ppm)	Relative Abundance (%)
Control	A2G0F/A2G0F	146,215.4	146,220.2	4.8	32.5	49.8
	A2G0F/A2G1F	146,374.6	146,382.3	7.7	52.3	36.7
	A2G1F/A2G1F	146,543.6	146,544.4	0.8	5.5	13.4
40 °C, 1 h	A2G0F/A2G0F	146,218.6	146,220.2	1.6	10.6	43.7
	A2G0F/A2G1F	146,375.9	146,382.3	6.4	43.7	40.1
	A2G1F/A2G1F	146,530.7	146,544.4	13.7	93.8	16.2
60 °C, 1 h	A2G0F/A2G0F	146,213.2	146,220.2	6.9	47.4	50.7
	A2G0F/A2G1F	146,376.8	146,382.3	5.5	37.4	33.6
	A2G1F/A2G1F	146,543.1	146,544.4	1.3	9.1	15.8
FTC 1	A2G0F/A2G0F	146,214,.8	146,220.2	5.4	37.0	51.0
	A2G0F/A2G1F	146,374.2	146,382.3	8.1	55.4	30.5
	A2G1F/A2G1F	146,535.1	146,544.4	9.4	63.8	18.4
FTC 5	A2G0F/A2G0F	146,217.6	146,220.2	2.6	17.8	49.9
	A2G0F/A2G1F	146,379.4	146,382.3	2.9	19.7	33.8
	A2G1F/A2G1F	146,539.1	146,544.4	5.3	36.2	16.4
Agitation 24 h	A2G0F/A2G0F	146,215.9	146,220.2	4.3	29.3	50.5
	A2G0F/A2G1F	146,375.1	146,382.3	7.2	49.3	31.0
	A2G1F/A2G1F	146,541.1	146,544.4	3.3	22.6	18.5
Light 24 h	Not identified	146,243.9	-	-	-	66.6
	Not identified	146,428.2	-	-	-	33.4

**Table 6:** Overall results for experimental retention time and relative proportions in the SEC analysis. Results that are significantly different from the control appear in bold type.

Stress	Monomer		Dimer	
	Retention Time (min)	Proportion (%)	Retention Time (min)	Proportion (%)
Control (10 mg/mL)	8.70	99.6	7.84	0.4
40 °C 1 h	8.71	99.6	7.84	0.4
60 °C 1 h	8.83	99.7	7.90	0.3
FTC 1	8.60	99.5	7.80	0.5
FTC 5	8.73	99.6	7.90	0.4
Agitation 24 h	8.81	99.6	8.00	0.4
Light stress 24 h	8.84	**96.6**	7.60	**3.4**
Control (2 mg/mL)	7.96	99.5	6.87	0.5
NaCl	7.95	99.8	6.83	0.2

**Table 7:** Characteristic parameters of the standard calibration curve (calibration model) and the figures of merit of the ELISA method.

Parameter	Value
Mathematical model fitted	Polynomial model
Function	Y = −0.0002x^2^ + 0.0322x + 0.0387
s ^a^	0.0000087
s ^b^	0.0008737
s ^c^	0.0121092
R^2^ (%)	99.34
*p*-value	0.0252

^a^ Standard deviation of the square concentration coefficient; ^b^ Standard deviation of the concentration coefficient; ^c^ Standard deviation of the constant for the polynomial function.

**Table 8:** Accuracy and precision of the ELISA.

Nivolumab Concentration (ng/mL)	R (%) ^a^	RSD (%) ^b^	
		Intraday	Interday (3 Days)
25	99.2	6.16	4.75
10	106	8.53	3.67
5	92	10.24	11.96

^a^ Recovery value based on three determinations; ^b^ Relative standard deviation based on three determinations.

**Table 9:** Percentage estimation of the remaining activity in stressed nivolumab samples by ELISA.

Stress	Remaining Activity (%)
40 °C 1 h	94.2 ± 4.2
60 °C 1 h	90.2 ± 4.3
FTC 1	88.5 ± 4.1
FTC 5	91.9 ± 2.0
Agitation 24 h	80.2 ± 2.6
Light 24 h	69.2 ± 6.7
NaCl	91.8 ± 7.6

## Supplementary Materials

The following supporting information can be downloaded at: https://www.mdpi.com/article/10.3390/pharmaceutics14040692/s1, Figure S1: Resulting CD spectra of the temperature stability study by submitting nivolumab to a temperature ramp (from 20 °C to 90 °C); Figure S2: SE/UHPLC(UV) chromatogram for nivolumab fresh sample (Opdivo^®^ 10 mg/mL); Figure S3: Experimental size exclusion column calibration model; Figure S4: Standard calibration curve for the ELISA method.
