# Supplementary material for: Comprehensive Analysis of Nivolumab, A Therapeutic Anti-Pd-1 Monoclonal Antibody: Impact of Handling and Stress
Source: Pharmaceutics. 2022 Mar 23;14(4):692. doi: 10.3390/pharmaceutics14040692 (PMC9025134; doi:10.3390/pharmaceutics14040692)
Supplement: Supplementary file 1 [file pharmaceutics-14-00692-s001.zip › pharmaceutics-1609927-supplementary.pdf]

pharmaceutics

Supplementary Materials: Comprehensive Analysis of Nivolumab, A Therapeutic Anti-Pd-1 Monoclonal Antibody: Impact of Handling and Stress

Anabel Torrente-López , Jesús Hermosilla , Antonio Salmerón-García , José Cabeza and Natalia Navas

Circular dichroism

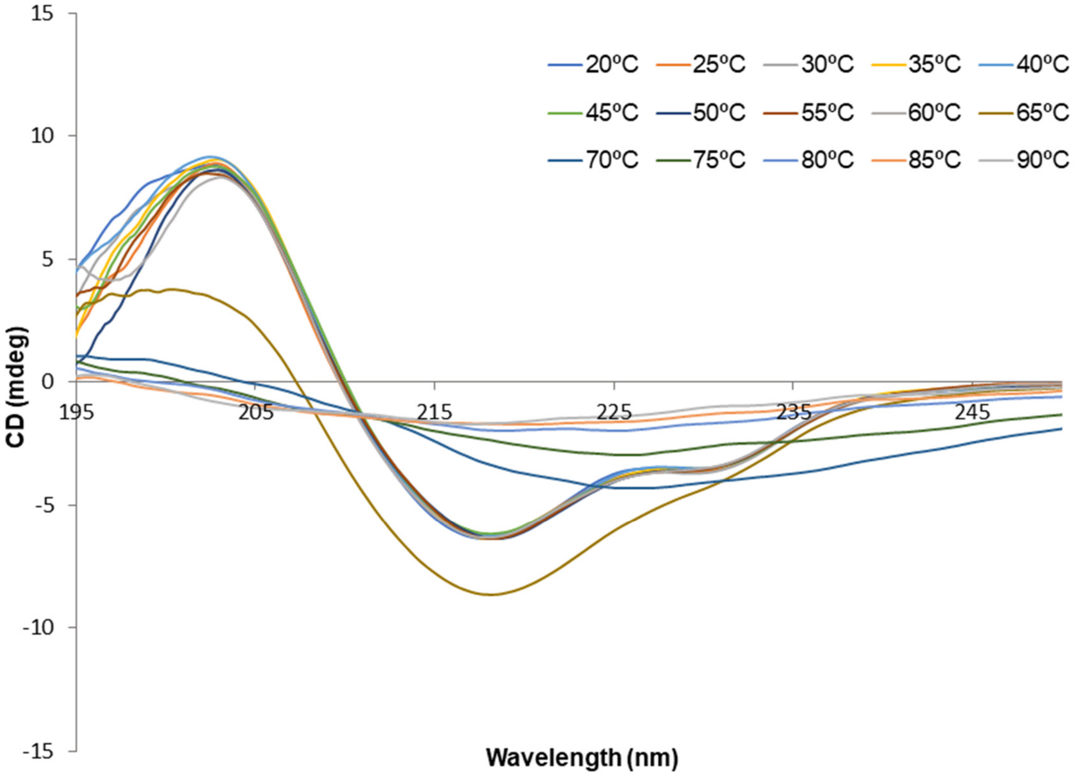

Figure S1. Resulting CD spectra of the temperature stability study by submitting nivolumab to a temperature ramp (from 20 °C to 90 °C).

Aggregation study by SE/UHPLC(UV)

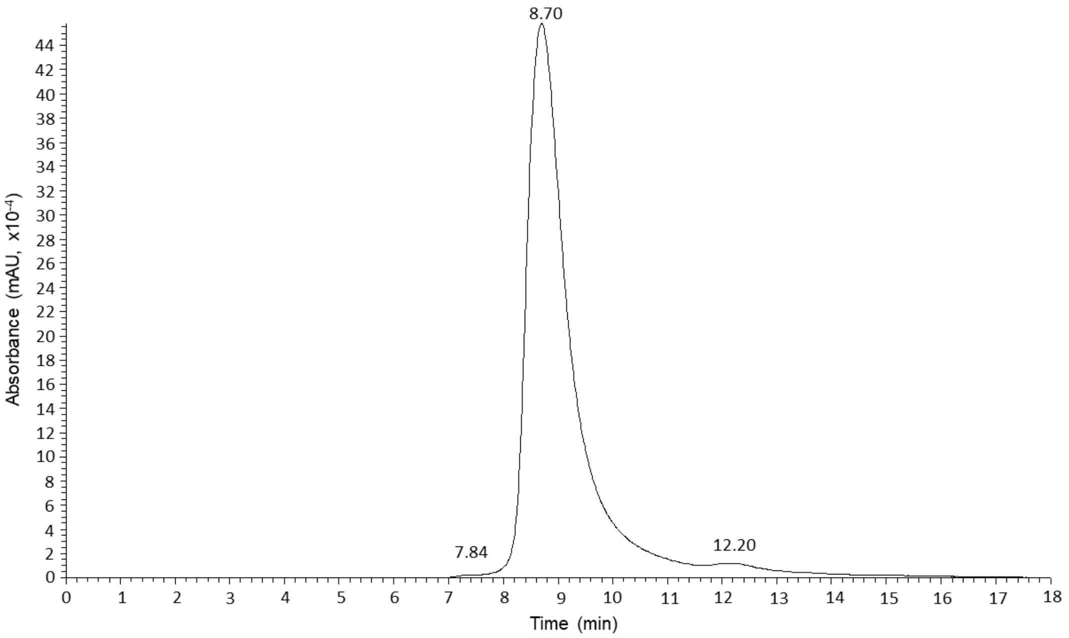

Figure S2. SE/UHPLC(UV) chromatogram for nivolumab fresh sample (Opdivo® 10 mg/mL).

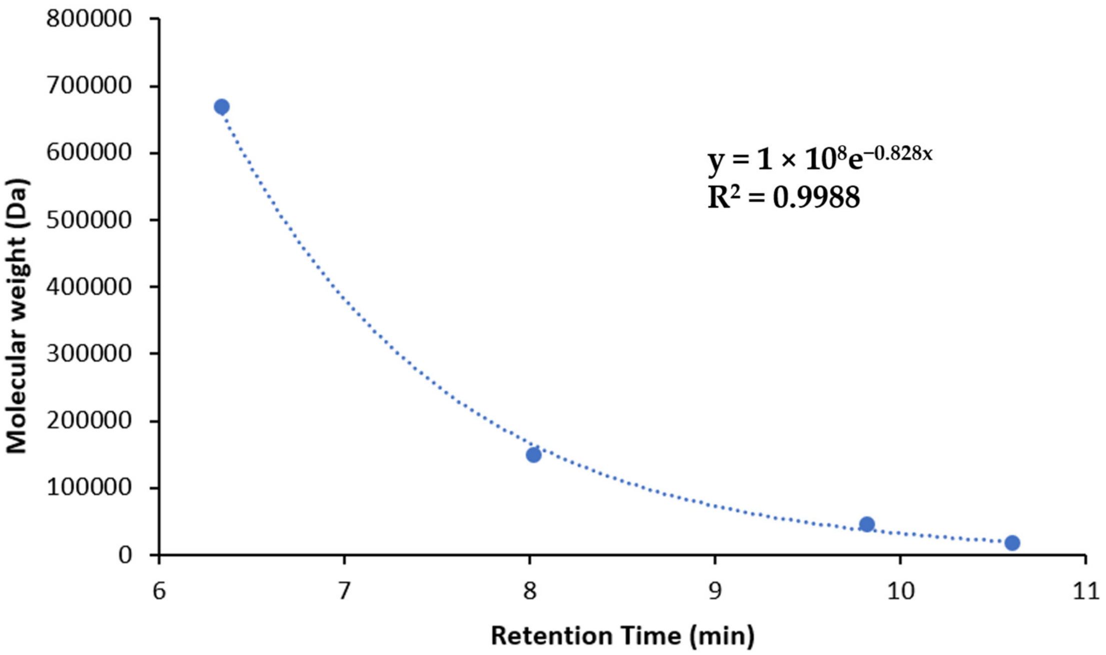

Figure S3. Experimental size exclusion column calibration model.

ELISA

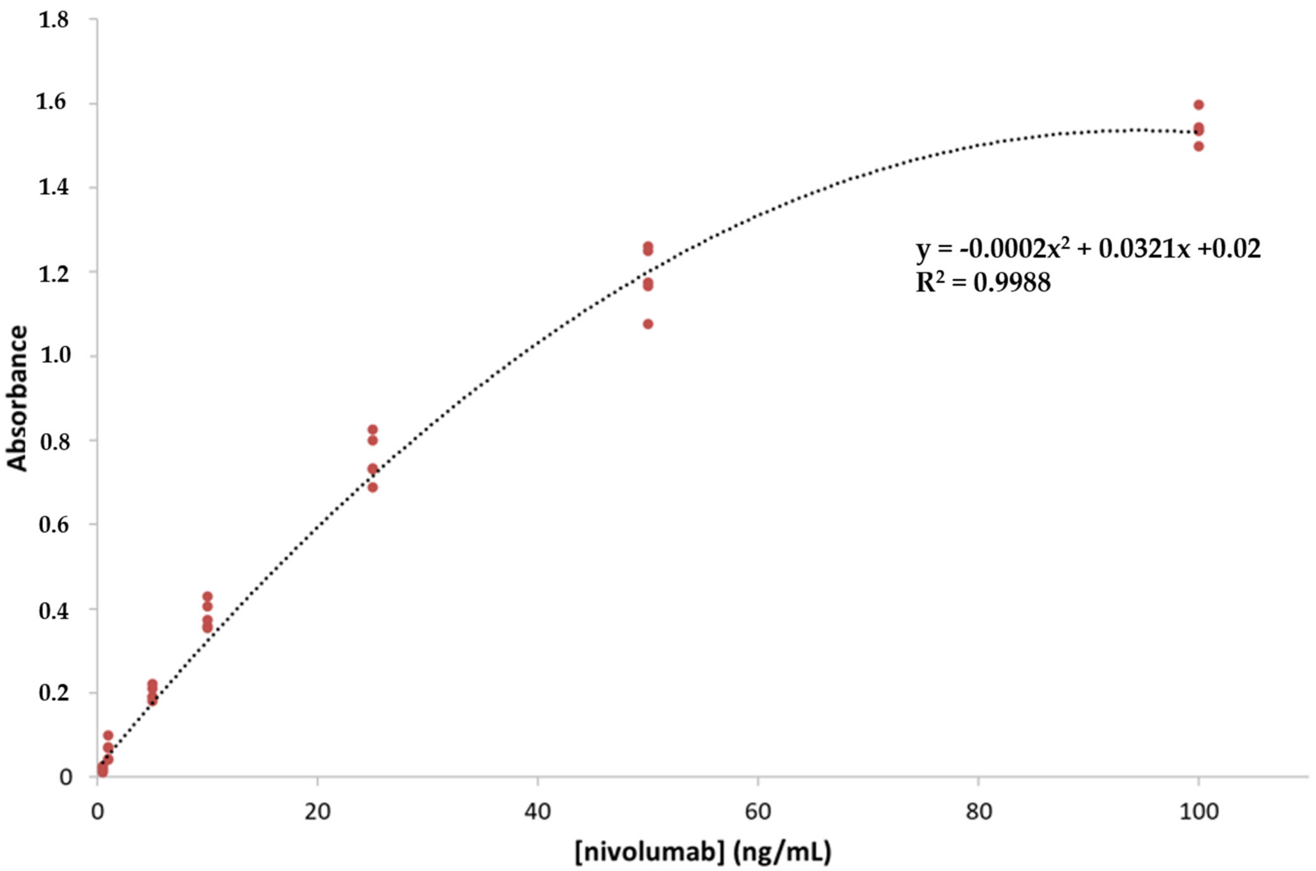

Figure S4. Standard calibration curve for the ELISA method.
